# Dietary Intake of Essential Elements from African Foods Available in the UK Market

**DOI:** 10.3390/foods15122171

**Published:** 2026-06-16

**Authors:** Eid I. Brima, Parvez I. Haris, Michael Frei

**Affiliations:** 1 School of Allied Health Sciences, Faculty of Health and Life Sciences, De Monfort University, Leicester LE1 9BH, UK; pharis@dmu.ac.uk; 2 Institute of Agronomy and Crop Physiology, Justus-Liebig-University, Heinrich-Buff-Ring 26–32, 35392 Giessen, Germany; michael.frei@agrar.uni-giessen.de

**Keywords:** essential elements, Africa, foods, health, dietary, UK, ICP-MS, hazard quotient, hazard index, excess index, micronutrient, malnutrition

## Abstract

Background: Foods containing essential elements are important to human health. There is concern regarding micronutrient deficiency in the African population, and there is a need to identify foods that can address this public health issue. This study focuses on the determination of essential elements (EEs) in four African food categories: plant-based agricultural products (PBAPs), spices (SPs), fishery products (FPs), and non-food items/additives (NFAs) on sale in the UK market. Methods: Inductively coupled plasma mass spectrometry (ICP-MS) was used for measuring trace essential elements (TEEs—Mn, Fe, Cu, Zn, Se) and major essential elements (MEEs—Na, Mg, K, Ca) in the four categories of the African foods. Results: Mean concentrations (µg/g) for the TEEs were Cu 5.3, 7.3, 23.5, and 7.4; Fe 79.0, 263, 107.7, and 1311.3; Mn 23.4, 28.5, 15.9, and 47.4; Se 0.3, 0.1, 1.6, and 0.6; Zn 8.9, 11.4, 26.8, and 6.2 (PBAPs, SPs, FPs, NFAs, respectively). Mean concentrations of the MEEs (mg/g) were Na 0.6, 11.2, 13.3, and 32.9; Mg 1.6, 1.9, 2.4, and 5.5; K 9.2, 14.6, 9.6, and 8.3; Ca 4.1, 3.3, 27.5, and 127.8. All elements were below the upper intake limits (ULs) established by WHO/EFSA. When expressed as a percentage of the recommended daily allowance (%RDA) for adult males/females, 100% or more of the requirement was achieved for Cu (107.1%) and Ca (112.8%) in FPs. Excess index (EI), hazard quotient (HQ) and hazard index (HI) values for all TEEs were <1, indicating there is no non-cancerogenic health concern across all food categories. Conclusions: These findings demonstrate that African foods available in the UK are substantial sources of essential micronutrients. The fishery products contain high levels of nutrients that are often deficient in African diets. However, before recommending these foods for nutritional interventions, a comprehensive risk–benefit assessment, considering potential toxic metal contamination and microbial hazards must be undertaken. Future studies should expand the sample pool to include a broader range of African foodstuffs and national representation, coupled with integrated risk-benefit analyses.

## 1. Introduction

The presence of essential elements (EEs) such as trace essential elements (TEEs) copper (Cu), manganese (Mn), iron (Fe), selenium (Se), zinc (Zn) and major trace elements (MEEs) sodium (Na), magnesium (Mg), potassium (K), and calcium (Ca) in food is important for public health. Both Cu and Zn are important as parts of the composition of some enzymes and their role in vital metabolic processes in humans. Zn is involved in over 300 enzymatic reactions, immune function, protein synthesis, and gene expression. Consequently, inadequate intake of Zn may result in impaired growth and immune dysfunction [1]. In addition, Cu plays a key role in iron metabolism, antioxidant enzyme systems, and neurological function [2]. Iron is an essential component of hemoglobin and myoglobin and is crucial for oxygen transport and mitochondrial respiration. Hence, iron deficiency remains one of the most prevalent nutritional disorders globally and is associated with anemia and impaired cognitive development. Mn is essential for bone formation, amino acid metabolism, and antioxidant defense, which protects cells from oxidative stress. Se is part of selenoproteins that regulate thyroid hormone metabolism and immune responses. Deficiency and excessive intake of Cu, Mn, Fe, Se, and Zn may impair human health. Therefore, adequate intake of the EEs is necessary for optimal health because both deficiency and excess may lead to adverse health effects. Consequently, TEEs are important dietary elements required for growth, immune competence, neuromuscular function, antioxidant, and cardiovascular regulation [2,3,4,5,6,7]. Although there are important roles for essential elements in biological systems regarding health in humans, excess intake of Mn has adverse health effects, such as reproductive and neurological dysfunction [8]. Zinc has a role in sperm, lipids, proteins, and nucleic acids synthesis, fetus development, and immune response [9,10]. However, Zn deficiency can impair reproduction in male-decreased serum testosterone, hyperammonemia, and decreased serum thymulin activity [11]. Se prevents the progression of cancer cells; hence, it is useful in cancer reduction. It is also useful in slowing aging by preserving the elasticity of the body and stimulating antibody formation. A recent study found that there was a strong association between Se deficiency and a high probability of febrile seizure [12,13].

Regarding MEEs, Mg is a cofactor in various enzymatic reactions, including energy metabolism and neuromuscular transmission. Moreover, Mg is important for cardiovascular health and glucose regulation. Na and K are crucial for fluid balance, nerve impulse transmission, and muscle contraction. Ca is fundamental for bone mineralization, muscle contraction, and blood clotting [14]. However, excessive Na intake is associated with hypertension and increased cardiovascular disease risk [14,15,16]. Generally, these MEEs are needed in ideal amounts to maintain optimal human health by contributing to metabolic equilibrium. Exposure to MEEs should be evaluated to avoid deficiency or excessive exposure, which could lead to adverse health concerns [5,15].

Due to the importance of the EEs in nutrition, a study [17] investigated essential elements (Na, K, Mg, Ca, Fe, Zn, Cu, and Mn) contents in different Nigerian dishes. Their study showed low levels of Fe and Zn and recommended consumption of fortified foods. Many studies have assessed elements in foods [18,19,20,21], especially for Cu, Fe, Mn, Se, Zn, Na, Mg, K, and Ca. The types of foods investigated were meat, fish, fruits, and vegetables. Agricultural foods are sources of essential elements, micronutrients, and vitamins [22,23]. Food grains and spices commonly used in Kenya were assessed for trace elements, including Se and Zn [24]. They were all agricultural products including coriander, ginger, garlic, cloves, lemon grass, rosemary, wheat, brown rice, finger millet, bulrush millet, sorghum, sunflower seeds, watermelon seeds, and pumpkin seeds. Se and Zn were reported to be the highest concentrations in Bulrush millet and pumpkin seeds, respectively. Their study concluded that the consumption of different foods of mixed diet may possibly provide the human body with essential trace elements, which lead to optimal health and boost immunity. Non-food items such as calabash clay (Nzu) and other types of geophagy materials are consumed in parts of Africa [25]. They can also be a source of essential elements. For example, one study determined the contents of essential elements including Na, Mg, K, Ca, Cu, Mn, Fe, and Zn in calabash clay [26]. They showed that the content of Ca, K, Mg, Na, and Zn did not exceed the recommended daily intake (RDI), while Fe exceeded the RDI.

To the best of our knowledge, this is the first study to investigate essential elements (Mn, Fe, Cu, Zn, Se, Co, Mo, Na, K, Mg, Ca) in African foods available for sale in the UK and those commonly consumed by African populations. It is a food monitoring study involving analysis of raw foods as purchased from the market. In a recent study, we reported the content of toxic elements for the same food samples [27]. A study by other researchers reported the content of essential elements in African and Caribbean dishes [28]. It was a total diet study (TDS) analysis of foods as consumed. The main aim of our research was to measure the levels of the EEs in African foods sold in the UK and determine the %RDA, EDI, EI, HQ, and HI. These assessments are crucial for nutrition adequacy of the essential elements in the investigated African foods. The study will also identify foods that can mitigate the deficiencies of essential elements in the African diet.

## 2. Materials and Methods

### 2.1. Sample Collection and Preparation

Four categories of African foods on sale in the UK market were collected between 2023 and 2024. The four food types were plant-based agricultural products (PBAPs), fishery products (FPs), spices (SPs), and non-food/additives (NFAs) that are consumed. The total number of the samples was 152. Categories and subcategories, common names, scientific names are presented in Table 1. All samples were powdered with a coffee grinder and then digested as described in the following section.

### 2.2. Digestion Method

A total of 0.3 g of a dried food sample was used for the digestion process. In total, 10 mL volume was used for the digestion process: 2 mL (H_2_O_2_, 30%),) and 8 mL (HNO_3_, 65%). The total time of the digestion process was 105 min: 5 min ramp step (1000 watts, 190 °C), 40 min hold step (1000 watts, 190 °C), and 60 min cooling step. The digested sample was made up to 25 mL with deionized water.

### 2.3. Analysis Method Using ICP-MS

Nine essential elements (Ca, Na, K, Mg, Cu, Fe, Mn, Se, Zn) were analyzed in all collected samples (*n* = 152) by using by Inductively Coupled Plasma Mass Spectrometry (ICP-MS), Agilent 7900 ICP-MS (Santa Clara, CA, USA). The operating conditions for the ICP-MS was same as described in [27].

### 2.4. Analytical Method and Chemicals

A multi-element Calibration Standard 2A (10 µg/mL) in 5%HNO_3_ from Agilent Technologies (Santa Clara, CA, USA) was used. Stock solutions for major essential elements were obtained from Thermo Fisher Scientific, Waltham, MA, USA, Na, Mg, and Ca (10,000 µg/mL) and K (1000 µg/L).

Calibration standards for trace essential elements (Cu, Fe, Mn, Se, Zn) were prepared at concentrations of 5, 10, 20, 40, and 80 μg/L by using 1%HNO_3_. Calibration standards for the major essential elements (Na, Mg, K, Ca) were prepared at concentrations of 5, 10, 20, 40, and 80 mg/L by using 1%HNO_3_.

Internal standards were prepared from stock solutions: 1006 ± 5 mg/L of scandium (Sc) standard for ICP, 1000 mg/L of rhodium (Rh), and germanium (Ge) were obtained from Sigma-Aldrich Chemie (Steinheim, Germany). Fresh calibration standards for analysis were prepared daily by diluting stock solutions in 1%HNO_3_. Sc of 100 μg/L, Rh, and Ge were used at a concentration of 20 μg/L.

### 2.5. Quality Control

The background signals and sensitivity of the ICP-MS were checked before starting the analysis of the samples, by using tuning solution from Agilent containing Li, Mg, Co, Y, and Tl with concentration (1 μg/L) of each element in 2%HNO_3_. Additionally, helium (He) gas was used. Limit of detection (LOD) was determined for each measured element by the software (MassHunter 4.2 version C.01.02 Workstation), then limit of quantification (LOQ) was calculated as follows: LOQ = 3.33×LOD. The LODs and LOQs (μg/L) as follows: Cu (0.107, 0.321), Fe (0.909, 2.727), Mn (0.114, 0.342), Se (0.145, 0.435), and Zn (0.238, 0.793). For major elements the LODs and LOQs (mg/L) are as follows: Na (0.001,0.003), Mg (0.001, 0.003), K (0.015, 0.5), Ca(0.026, 0.087).

The quality control (QC) was assessed by using continuing calibration verification (CCV) for each batch. A standard 40 μg/L of a mixed standard of trace essential elements was measured four times within each batch. The QCs recoveries in one batch were as follows: Mn (96.1%), Fe (96.1%), Cu (94.9%), Zn (97.6%), Se (97%). A standard 40 mg/L of as mixed standard of major essential elements was measured four times within each batch. The QCs recoveries in one batch were as follows: Na (90.6%), Mg (90.7%), K (98.1%), Ca (102.1%).

### 2.6. Quality Assurance (QA)

The accuracy of the measurement was determined by measuring Standard Reference Material 1568c Rice flour and ERM-BB422—fish muscle.

In the Standard Reference Material 1568c Rice flour, the certified (mg/kg) and measured (mg/kg) values were as follows: Cu (3.06 ± 0.18; 2.59 ± 0.01), Fe (13 ± 1.5; 11.14 ± 0.04), Mn (30.92 ± 0.22; 23.88 ± 0.51), Se (0.064 ± 0.0023; 0.0695 ± 0.002), Sr (0.483 ± 0.01; 0.002 ± 0.01), Zn (23.45 ± 0.16; 16.05 ± 0.05).

The values for certified (mg/kg) and measured (mg/kg) in the ERM-BB422—Fish muscle were as follows: Cu (1.67 ± 0.16; 1.89 ± 0.001), Fe (9.4 ± 1.4; 7.8 ± 0.01), Mn (0.368 ± 0.028; 0.342 ± 0.003), Se (1.33 ± 0.13; 1.38 ± 0.01), Zn (16 ± 1.1; 12.5 ± 0.03). Certified (g/kg) and measured (g/kg) values for the major essential elements were as follows: Na (2.8, 2.6 ± 0.002), Mg (1.37, 1.39 ± 0.003), K (21.4, 19.5 ± 0.01), Ca (0.342, 0.316 ± 0.0005).

Additionally, spiked samples were used for QA. For trace essential elements (Cu, Fe, Mn, Se, Zn) elements a mix of, 50 µg/L of each element was spiked in a sample, and the recoveries were as follows: Mn (89.9.8%), Fe (86.8%), Cu (88.8%), Zn (99%), Se (87.1%). For major essential elements a mix of 50 mg/L of each element was spiked in a sample, and the recoveries were as follows: Na (99%), Mg (99.9%), K (94.6%), Ca (101.2%).

### 2.7. Calculations Procedures

#### Calculation of the Estimated Dietary Intake of Elements (EDIs) and Hazard Quotients (HQs)

The consumption of every food type among the four categories, plant-based agricultural products, fishery products, spices, and non-food items/additives was based on continental averages from FAOSTAT food balance categories and different African dietary studies g/day [29,30,31,32]. Therefore, the EDIs for trace essential elements (TEEs) measured in this study (Cu, Fe, Mn, Se, and Zn) were calculated by using the following steps and equations. The daily consumption per gram for spices (1.24 g), leaves (0.65 g), and fruits (0.95 g) was calculated as an average from [29]; tuber crops (37.8 g), seeds (146.9 g), and legumes (26.8 g) were established by a total diet study among sub-Saharan countries [30], and fishery products for dried fish (41 g) was established by [31]. No acceptable daily intake (ADI) has been allocated for Gum Arabic. However, according to the European Food Safety Authority (EFSA), 30 g is considered safe for daily intake [32]. There is no literature data available regarding the precise intake of Kaun (potash) or Chuna (Calcium Hydroxide). However, it is often stated that a pinch of these substances is used. Therefore, we took a pinch of Chuna and a pinch of Kaun and weighed them to determine the quantity in grams. This equated to 0.5 g for Chuna and Kaun. Chuna is consumed with areca nuts and *Piper betel* leaves, and Kaun is used in cooking. For calabash chalk-Nzu, which is consumed as part of geophagy practice, a reported value of 20 g was used [25].

For the first step, we calculated the daily intake (DI) as follows:

(1) DI = [D (g) × C (mg/g)]/1000

where DI unit is mg/day, D in g established by FAO or calculated by different publications, which is the assumed consumed food per day, where the equation is divided by 1000 to convert from µg to mg. In the case of MEEs, there is no need to divide by 1000, because the concentration is already expressed in mg/g.

For the second step, EDIs were calculated as follows:

(2) EDI = DI/BW

where EDI unit is mg/kg bw day, BW is body weight, which is considered to be 70 kg.

For the final and third step, hazard quotients (HQs) EDIs were calculated as follows:

(3) HQ = EDI/PMTDI

where HQ is hazard quotient (unitless), and PMTDI is provisional maximum tolerable daily intake (mg/kg bw/day).

The PMTDIs are Cu (0.5), Zn (1), and Mn (0.36) [33], Fe (0.8) [34], Se (9.4 µg/kg bw/day = 0.0094 mg/kg bw/day) [35].

(4) HI = ∑HQi

where HI is hazard index, which is the summation of all HQi of all trace elements (TEEs) in one food type, where i represents Cu, Fe, Mn, Se, and Zn. When HQ or HI is <1 it is considered safe, and when it is ≥1 it is considered a potential risk.

Calculation of the percentage recommended daily allowance (%RDA) and Excess index (EI).

The %RDAs were calculated for TEEs and MEEs. For MEEs there is no established PMTDI. Therefore, only the %RDAs for measured MEEs (Na, Mg, K, and Ca) in this study, the %RDAs were calculated by using the following equation:

(5) %RDA = ((EDI×BW)/RDA) ×100

where RDA is the recommended daily allowance (mg/day). Importantly, EDI must be converted to mg/day by multiplying it by the BW and then divided by RDA (mg/day). When %RDA < 100, ≈100, and >100, the intake is below requirement, meets recommendation, and exceeds recommendation, respectively.

The RDA (mg/day) for Cu (900, 900 µg/day), Mn (2.3, 1.8), Fe (8, 18), Se (55, 55 µg/day), Zn (11, 8), for men and women respectively [36]. While the RDA (mg/day) for Mg (400, 310), Ca (1000, 1000), and adequate intake (AI) Na (1500, 1500), K (3400, 2600), for men and women, respectively [3,14,15].

(6) EI = (EDI (mg/Kbw day) × BW (kg bw))/UL (mg/day)

where EI is the excess index (unitless), UL is the tolerable upper intake level (mg/day).

An EI > 1 indicates that the dietary intake exceeds the safe upper limit.

## 3. Results and Discussions

Table 2 shows that, among the four food categories (plant-based agricultural products, spices, fishery products, and non-food items/additives consumed), the highest concentrations (µg/g) of Cu, Fe, Se, and Zn were found in fishery products, while the highest concentrations of Mn were found in spices. In the 12 subcategories (leaves, tuber-crops, seeds, fruits, legume, Gum Arabic (*Acacia senegal*), spices, fish, prawns, Kaun (potash), calabash chalk-Nzu, and chuna (slaked lime mainly composed of calcium hydroxide), the highest concentrations of Cu and Se are in prawns, Fe in calabash chalk-Nzu, Mn in leaves, and Zn in fish. Our findings are consistent with [37], who reported the highest concentrations of Cu and Zn in prawns and fish, respectively.

Cu and Zn concentrations (µg/g wet weight) were reported in different fish species by [38], which ranged from 0.31 to 12.08 for Cu and 4.51 to 19.21. These values are close to our mean concentrations (µg/g dry weight) 9.4 and 29.5 for Cu and Zn in fish, respectively. A study [39] reported mean concentrations (mg/kg) of Cu, Mn, and Zn in calabash chalk-Nzu, where the Cu (15.5) level is similar to the average level of our results presented in Table 2.

Table 3 shows that no element in the subcategory foods exceeded the upper intake limit (UL) guideline value. Moreover, only Se and Zn do not show any values that exceeded the RDI for all subcategory foods. However, there are some elements in different foods that exceeded the recommended daily intake (RDI) values. The exceeded elements were found to be Cu and Se in prawns, and Fe in calabash chalk-Nzu. This shows that the following elements exceeded 50% of the RDI: Cu, Fe, and Mn in seeds; Mn and Se in fish; and Fe in prawns.

Table 4 shows that the highest calculated percentages of the recommended daily allowance (%RDA) for the trace essential elements (TEEs) exceeding 100% was only found in fishery products for Cu; the other four elements (Fe, Mn, Se, and Zn) were the highest %RDA among the four food categories. In the 12 subcategories, the highest calculated %RDA exceeded 100% were for two elements (Cu (171.3%), Fe (138.9%)) in prawns and calabash chalk-Nzu, respectively. The highest %RDA among elements were Cu (76.7%), Fe (58.2%), Mn (72.2% and 92.2% male and female, respectively) in seeds. Mn (59.5%) in fish, Fe (66.4%) in prawns, and Fe (61.7%) in calabash chalk-Nzu.

The excess index (EI) was calculated by dividing EDI/UL to estimate the overconsumption of the TEEs in the four categories of food types. All TEEs in all food categories were EI < 1. Therefore, no expected adverse health effects may result from consumption of these foods with exposure to the tolerable upper intake level (UL).

**Figure 1:**
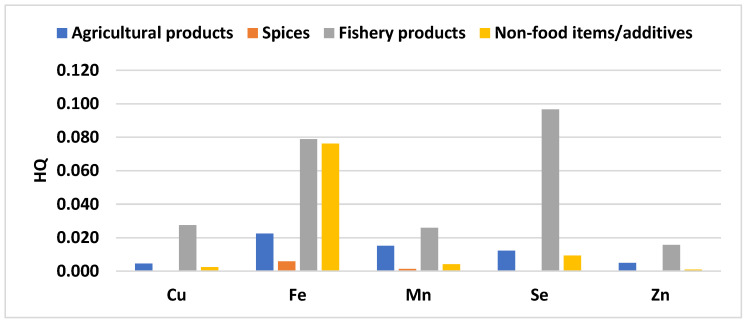
The HQs of Cu, Mn, Fe, Se, and Zn for the four food categories: plant-based agricultural products, fishery products, spices, and non-food items/additives.

Figure 1 shows that among the four food categories no one food type exceeded the guideline values, all HQ values were <1. The hazard index (HI) was calculated, and all HI were also <1.

Table 5 demonstrates that, among the four food categories (agricultural products, spices, fishery products, and non-food items/additives), the highest concentrations (mg/g) were Na (32.9), Mg (5.50), and Ca (127.8) in non-food items/additives, and K (14.5) in spices.

In the 12 subcategories (leaves, tuber-plant, seeds, fruits, legume, Gum Arabic, spices, fish, prawns, Kaun, calabash, and chuna, the highest concentration (mg/g) of Na (98.5), Mg (9.4) was found in kaun, K (17.2) in leaves, and Ca (36.4) in chuna, because chuna is a paste of Ca(OH)_2_ in water. The latter was measured after drying the sample overnight in the oven (4.18% moisture). Therefore, the percentage of Ca in Ca(OH)_2_ is 54.1% (541 mg/g) in wet weight and 56.41% (5641 mg/g) in dry weight. Chuna is a non-food item that is ingested after combining it with areca nuts and *Piper betel* leaves. Prawns were reported to have the highest concentration of Ca (39.7) among the rest of the subcategory foods.

Table 6 shows that no major essential element (MEEs) in subcategory foods exceeded the upper intake limit (UL) guideline value. Moreover, only Ca (1627.1 mg/day) among the MEEs was higher than its RDI in prawns.

Table 7 shows that the highest calculated percentages of recommended daily allowance (%RDA) of the major essential elements (MEEs) exceeding 100% was found only for Ca (112.8%) in fishery products. In the 12 subcategories, the highest calculated %RDA exceeding 100% were only for Ca (162.7%) in prawns. By calculating the excessive index (EI), no elements exceeded > 1, which indicates no health harm is expected from the consumption of prawns. It is noteworthy that fish and prawns contain the highest levels of Na. This could pose health risks for those who have a high intake of fish and prawns.

**Figure 2:**
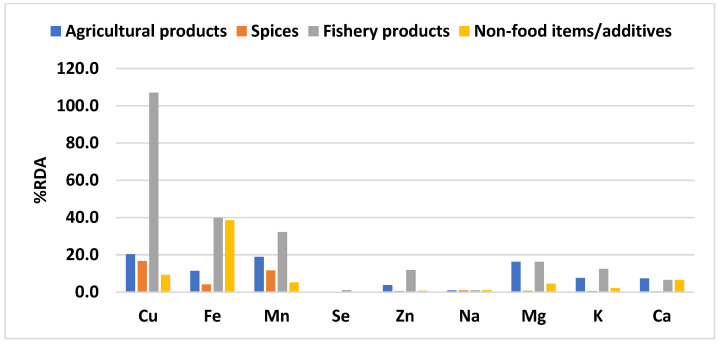
The %RDA of Na, Mg, K, Ca, Cu, Mn, Fe, Se, and Zn for the four food categories: plant-based agricultural products, fishery products, spices, and non-food items/additives.

Figure 2 shows only one food category that scored higher than 100% for the %RDA of Cu. Three out of the four food categories scored in the range 23.1 to 39.8% of the RDA for both TEEs and MEEs (Cu, Fe, Mn, and Ca). The rest were in the range between 0.1 to 18.2%. Therefore, we can conclude that some of the investigated African foods in this study are rich in essential elements and can be an appropriate source of the essential elements (EEs). More caution should be taken regarding the consumption of high amounts of some fishery products, spices, and PBAPs to avoid overexposure to EEs.

### 3.1. Plant-Based Agricultural Products (PBAPs)

In a previous study [40], contents of selected elements, including Na, Mg, K, Ca, Cu, Fe, and Zn, were determined in green and roasted coffee. The levels of these elements showed higher concentrations in roasted coffee than in green coffee, except for Cu and Fe. The latter study concluded that their levels were safe for health. Their results showed Cu level lower than Fe, and Na was the lowest level among the MEEs, which is similar to our results for seeds (Table 2), but not the same results. This is understandable because different seeds were investigated in our study, and it did not include coffee. Furthermore, the seeds may have originated from different geographical locations and may have been grown in different agricultural conditions.

In another study [41], eleven food groups were investigated for Cu, Fe, Mn, and Zn, including agricultural products and animal products. They calculated the estimated daily intake (mg/person/day) 2.7, 3.7. 8.7, and 22.3 for Cu, Mn, Zn, and Fe. This is similar to the trend of our results, Cu (0.2), Mn (0.4), Zn (0.4), and Fe (1.3), with the lowest level for Cu and the highest for Fe. They concluded that all measured elements were within international recommendations and therefore suggested that these analyzed foods were safe for consumers [41]. However, they also reported that some of the participants (0.5%) exceeded the UL for Fe. In our case, no element (Cu, Fe, Mn, Zn) exceeded the UL or was higher than the RDI.

The following essential elements: Na, Mg, K, Ca, Cu, Fe, Mn, Se, and Zn were determined in a total diet study among African and Caribbean dishes, snacks, and beverages consumed in the UK [27]. The latter study reported only a few of the EEs in the investigated foods and beverages. The study reported that their data will allow for better quantification of nutrient intake, and health professionals can give appropriate dietary advice and identify which foods can be encouraged for consumption. The increasing order of the MEEs reported in their study is as follows: Mg < K < Na < Ca, which is the same trend in our study in fishery products, presented in Table 5. However, the TEEs do not show a similar trend to our study. Both studies agreed on the presence of the investigated EEs in African foods.

### 3.2. Fishery Products

A study [31] reported elemental composition levels (µg/g) in different fish species from Africa (the Gulf of Guinea). Their results for Cu (12.1) and Zn (19.2) are similar to our results for Cu (9.4) and Zn (29.5). They recommended that the consumption of such species (*shrimp P. notialis*) should be avoided due to health concerns. In addition, by calculating EDI from our results in Table 3, prawns showed a high Cu concentration (1.5 mg/day), which exceeded the RDI as well. Our conclusions are similar to their recommendations that cautious consumption of the types of prawns investigated in their study should be taken into consideration due to the high EDI of Cu, which exceeded the RDI.

Four essential elements (Cu, Mn, Se, and Zn) were determined in fish and shellfish, where the highest level was reported for Cu in prawns and shrimps, and the highest level for Zn was reported in fresh fish and canned muscle [37]. A similar trend is seen in our results, where Cu was the highest among the four elements in prawns, and Zn was the highest in fish.

In a pervious study, the elemental composition of swordfish was determined in [42]. They detected the concentrations of Zn and Cu ranged from 3.4 to 15.7 µg/g and 0.3–1.9 µg/g respectively. In our case, for fish the concentrations for Zn and Cu were (0.1–77.8 µg/g) and (0.4–32.3 µg/g), respectively (Table 2). This shows the same trend as their results, because Zn levels were higher than Cu levels. Moreover, copper and zinc in four freshwater fish species from Greece reported Zn levels were higher than Cu levels [43]. Likewise, a similar trend was also seen for tropical Wetland fish species from India and some fish species from the Caspian Sea, Iran [44,45].

### 3.3. Spices

A previous study analyzed the content of Se and Zn and other elements in different spices [24]. The concentrations of Zn 0.1–40.4 µg/g (mean 11.5 µg/g) and Se 0.06–0.3 µg/g (0.14 µg/g) show a similar trend as found in our study. Another study [46] determined the contents of essential elements (Na, K, Cu, Fe, Mn, Se, and Zn) in 20 spices. They concluded that six of the commonly used spices contributed 7.5% of the daily dietary intake of Fe, Mn, Cr, and Zn. The increasing order of the concentrations of the TEEs in their study was as follows: Se < Cu < Zn < Mn < Fe, which is the same as the order in our study (Table 2). They reported a higher K concentration than Na, which is also the same as our results (Table 5). The following essential elements (Na, K, Ca, Mn, Fe, and Zn) were determined in different Algerian spices [47]. The highest mean concentrations for K, Ca, Na, Fe, Mn, and Zn were detected in mint, oregano, fennel, verbena, black pepper, and coriander. In our study, the highest concentrations were found to be Fe > Zn in coriander (*Coriandrum sativum*) and chili powder-mitmita (*Capsicum frutescens*), while Fe > Mn> Cu > Zn in Banga spice (*Tetrapleura tetraptera*), Fe > Mn > Zn > Cu in Fasika spice (mixture of different spices), African pepper (*Capsicum frutescens*), and fennel (*Foeniculum vulgare*).

### 3.4. Non-Food Items/Additives

Essential elements, including K, Ca, Mn, Fe, Cu, and Zn, were previously determined in calabash chalk-Nzu [32]. Their study showed the following increasing trend: Cu < Mn < Zn < Ca < K < Fe. This shows a similar trend to our results, and almost the same levels (mg/kg) for Cu and Ca (Table 2 and Table 5). Consumption of calabash chalk-Nzu as part of a geophagy practice is risky due to the presence of toxic elements [25]. Although the calabash chalk-Nzu can be a source of some essential elements, the potential risks may outweigh the benefits. Kaun (Trona) has been previously investigated for elemental composition (Na, Mg, K, Ca, Cu, Mn, Fe, and Zn) and its toxic impact on rats [48]. The authors of the study concluded that Kaun ingestion had a potential hepatotoxic effect on rats [48]. The same elements were also determined in our study, with the highest level of Na, which is the same trend as reported in their study. The increasing order of the trace elements in their study is as follows Zn < Mn < Cu < Fe, which is similar to our results. In another study [49], Trona (kaun) was administered to Wistar rats, which indicated that at low doses it can be a source of antioxidants. However, with higher doses up to 450 mg/kg it was found to be harmful to rats. The adverse health effects reported by the authors were as follows: a decrease in fertility hormones and semen parameters, causing prostate problems, and hypogonadism. They concluded that humans may have similar adverse health impacts due to the consumption of Kaun.

### 3.5. Essential Elements Deficiency in the African Diet and Suggested Foods to Mitigate the Deficiency

Some essential elements are low in African diets due to low levels of elements in soil being available in plant-based diets, low levels of such elements in foods consumed, and high phytate levels, which bind the metals and prevent their bioavailability. It is important to stress that the total content of each element measured by ICP-MS in our study does not necessarily reflect the amount that can be absorbed by the body. Deficiencies in African populations, for Ca (54%), Zn (40%), Se (28%), Cu (1%), and Mg (<1%) have been previously reported [50]. There are regional differences, with North and West Africa having lower deficiency. Fe deficiency was reported in the range from 9% to 18% among women in Ethiopia, Kenya, Nigeria, and South Africa [51] and up to 34.2% among children in the following African countries: Kenya, Uganda, Burkina Faso, South Africa, and Gambia [52]. Fe deficiency was also reported [50] to be between 5% and 43%, generally in Africa. Se deficiency was estimated to be 28% across Africa [53], with the following percentages in different regions: 52%, 49%, 26%, 12%, and 6% in East, Middle, South, North, and West, respectively.

In our study, we identified different food categories containing elements (Cu, Fe, Mn, Se, and Zn) that had high %RDA. Cu > 100%RDA in prawns, other elements showed >50%RDA in foods, seeds (Cu, Fe, Mn), fish (Mn), prawns (Fe), calabash chalk-Nzu (Fe). We identified that fishery products showed the highest levels of all four elements (Cu, Fe, Se, and Zn). Spices showed the highest content of Mn. Our findings are similar to a recent study [31], which reported that dried fish contributed to 15%RDA for six African countries (Ivory Coast, Ghana, Nigeria, Malawi, Tanzania, and Uganda), and it was found to be a good source of Se, Fe, Zn, and Ca.

From our study, we identified the following foods that can potentially mitigate the dietary deficiencies of essential elements in the African diet.

#### 3.5.1. Plant-Based Agricultural Products (PBAPs)

Seeds can be a very good source of Cu, Fe, and Mn. Prawns and calabash Chalk-Nzu can provide more than 50%RDA for Fe. Seeds showed high contents of these three elements among the 12 subcategory foods analyzed. There are six types of leaves (Ewedo (*Corchorus olitorius*), Okasi (*Gnetum africanum*), Mutsine (*Bidens pilosa*), Ugu (*Telfairia occidentalis*), herbal tea (*Lippia javanica*), bitter leaves (*Vernonia amygdalina*)) that were high in Mn (75.1 to 440.4 µg/g) and Fe (120.6 to 937.2 µg/g). The other two leaves, Kawal (*Cassia obtusifolia*) and Waake leaves (*Sorghum bicolor*) showed high Fe levels of 517.3 and 298.6 µg/g, respectively. Kawal (*Cassia obtusifolia*) is a fermented leaf and is considered an underutilized food in Darfur, Sudan. Kawal is considered a protein source and a meat substitute that also has high Ca content [54]. Our results also show that Kawal had the highest level of Ca (26 mg/g) among the foods analyzed. Hibiscus calyx (*Hibiscus rosasinensis*), which is neither leaves nor fruit, showed high average levels for Fe (240.6 µg/g) and Mn (470.5 µg/g). Hibiscus is often used as an herbal tea, recognized for its red color, and it can be consumed cold or hot. Among tuber crops, yam (*Dioscorea*) flour had the highest Mn (45.7 µg/g) content. From the spices category, ground fennel (*Foeniculum vulgare*) had the highest Fe (843.2 µg/g), and control onions (*Allium cepa*) had the highest average Mn (66.5 µg/g).

#### 3.5.2. Fish and Prawns

Koobi fish from Ghana, which is salted and sun-dried tilapia, had the highest average Cu (31.9 µg/g), Fe (141.3 µg/g), Mn (206.2 µg/g), and Se (3.4 µg/g). Our results in this case agree with the findings of a previous study [31], which reported dried fish in Africa is a good source of essential elements (Fe, Se, and Zn) and other micronutrients, which can play an important role in food security and nutrition. They stated that the nutritious benefits from dried fish were always overlooked, and it is also people’s preference to eat dried fish over fresh fish by up to 54%. We agree with the latter study that dried fish is a good source of essential elements, as demonstrated in our results, especially for Koobi fish (dried Tilapia). However, it is necessary to be cautious so that the daily amount consumed does not lead to exceeding the RDA. It is important to stress that in our previous study, we reported that dried and smoked fish also contained Pb and Cd that could pose health risks [27]. Therefore, it is important to consider whether the risks outweigh the benefits of eating dried fish. The process of drying and smoking fish can introduce toxic elements like lead and cadmium from the environment. It is also well known that dried fish often contain foodborne pathogens that can be harmful to human health [55]. Therefore, even though there is a preference for eating dried fish in Africa, the need to consider potential chemical and/or biological risks needs to be considered as part of food safety evaluations. In this context, we would recommend people to be cautious about consuming high quantities of dried fish to address deficiencies in essential elements.

Dried ground prawns had the highest average Fe (193.1 µg/g) and Se (2.1 µg/g), while whole smoked prawns contained Cu (41.2 µg/g), Fe (108.3 µg/g), and Se (1.8 µg/g). Copper (<30 mg/kg) was reported to be found in prawns’ gill [56] with other elements such as Zn and Cd (muscle and tissue); however, they are all below the permissible levels. Another study [37] reported high concentrations of Cu in shellfish-crustaceans (prawn and shrimp) due to hemocyanin (a copper-containing protein) [57], they determined Ca, Mg, Na, and K in the fish species of Chanoga and concluded that these fish species can provide mineral intake and combat mineral deficiency diseases.

#### 3.5.3. Non-Food Items/Additives

Calabash chalk-Nzu showed the highest average concentration for Fe (556 µg/g) and Se (0.9 µg/g). Although calabash showed the highest essential elements levels, it also contains high concentrations of toxic elements (As and Pb), as shown in our previous study [27] for the same samples. It was reported that As and Pb levels exceeded safety thresholds. Therefore, consumers should be careful because of the potential toxicity effects of As and Pb in calabash.

Our results (Table 6) demonstrated that only Ca among the MEEs exceeded the RDA in prawns. A BitekuTeku leaf (*Amaranthus* spp.), among leaves as a subcategory of food, had the highest average concentration (2.73 mg/g), coriander (7.3 mg/g) among spices, dried fish powder (made from different fish species) from Ghana (32.5 mg/g) had the highest Ca concentration among fish, and whole smoked prawns (40.3 mg/g).

Based on our above discussion, it is demonstrated that no single food can provide all the essential elements. However, fish is one of the foods that contains high levels of Fe, Mn, Cu, and Se. Diversification of foods consumed is encouraged to provide a balanced diet and combat micronutrient deficiency in African foods.

## 4. Conclusions

This study found that some African foods on sale in the UK, categorized into four groups (plant-based agricultural products (PBAPs), spices (SPs), fishery products (FPs), and non-food items/additives (NFAs), contain high concentrations of essential elements. Some essential elements (Cu and Ca in prawns), and Fe in calabash chalk-Nzu exceeded the 100%RDA. However, no food type shows a hazard quotient or hazard index > 1. Therefore, we concluded that the investigated African foods in this study can be a good source of essential elements. Both MEEs and TEEs are lower than the UL set by WHO and/or EFSA, while only Ca exceeded the RDI for FPs. This study establishes a provisional baseline and data for essential elements in some African foods, which will be important for future insight into such studies.

We identified different foods that can potentially combat some elemental deficiencies in the African diet. The identified foods that can provide a good source of essential elements in the diet are different leaves (Ewedo (*Corchorus olitorius*), Okasi (*Gnetum africanum*), Mutsine (*Bidens pilosa*), Ugu (*Telfairia occidentalis*), herbal tea (*Lippia javanica*), bitter leaves (*Vernonia amygdalina*), Kawal (*Cassia obtusifolia*), Waake leaves (*Sorghum bicolor*), Hibiscus (*Hibiscus rosasinensis*), control onions (*Allium cepa*), ground Fennel (*Foeniculum vulgare*) (Fe and Mn), Koobi fish-dried tilapia (Cu, Fe, Mn and Se), ground prawns, and whole smoked prawns (Cu and Se). It is well demonstrated that no single food can provide all the essential elements. Diversification of plant-based food and animal-based food is encouraged to provide a balanced diet and combat elemental deficiency in the African population. Some foods and non-foods, such as dried fish and calabash, can be a high source of certain essential elements. However, this should be considered along with information regarding the content of toxic elements and foodborne pathogens that are present in these products. Risk–benefit analysis, including food safety assessment, should be conducted before any type of food is recommended for human consumption. Larger studies that need to be conducted in the future include other categories of foods not covered in this study.

## Figures and Tables

**Table 1:** Categories of the African foods: Agricultural products (APs), fishery products (FPs), spices (SPs), and non-food additives (NFAs) and their detailed subcategories. Collected from different shops in different cities on sale in the UK markets.

Food Category	Food Subcategories	Common Names (Scientific Names)
Plant-based agricultural products (PBAPs)	Leaves	Cassava leaves (*Manihot esculenta* Crantz), Ewedo (*Corchorus olitorius*), Corn husk (*Zea mays*), Biteku Teku (*Amaranthus* spp., Okasi leaf (*Gnetum africanum*), Kawal-fermented leaves (*Cassia obtusifolia*), Mutsine (*Bidens pilosa*), Waake leaves (*Sorghum bicolor*), Red leaves (*Telfairia occidentalis*), Herbal tea (*Lippia javanica*), Bitter leaves (*Vernonia amygdalina*).
	Tuber-crops	Yam flour (*Dioscorea*), Cassava dough (*Manihot esculenta* Crantz), Control onions (*Allium cepa*), Bula-fakes banana (*Ensete ventricosum*).
	Seeds/cereals	White rice and brown rice (*Oryza sativa*), Teff (*Eragrostis tef*), Sorghum (*Sorghum bicolor* L.), village rice (*Oryza glaberrima*), whole white maize (*Zea mays*), corn dough masa (*Zea mays*), Sorghum flour (*Sorghum bicolor*), Banku dough fermented corn and cassava (*Zea mays* L.), Beso roasted barley (*Hordeum vulgare*), Ogbono (*Irvingia gabonensis*), whole Egusi (*Citrullus lanatus* var. *colocynthoides*), Pearl Millet (*Pennisetum glaucum* (L.) R. Br.), Millet (*Pennisetum glaucum*).
	Fruits	Dates Barakawe and Dates Gundil (*Phoenix dactylifera*), Tamarind (*Tamarindus indica*), Okra-frozen whole okra and Okra-powder (*Abelmoschus esculentus*), Baobab (genus *Adansonia*), Lalob (*Balanites aegyptiaca*), Masau (*Ziziphus mauritiana*), Limu (*Citrus aurantifolia*), Nabag (*ziziphus*).
	Legumes	Black eye beans (*Vigna unguiculata*), red skin peanut (*Arachis* *hypogaea*), red lentils (*Lens culinaris*), Ofor (*Detarium microcarpum*), Cow peas (*Vigna unguiculata*), brown lentils (*Lens culinaris*), ground peanut (*Arachis hypogaea*).
	Others	Gum Arabic is a plant exudate (*Acacia senegal*), Hibiscus is a Calyx (*Hibiscus rosasinensis*).
Fishery products (FPs)	Fish	Sardine Morocco (*Sardina pilchardus*), Aswen fish (*Oreochromis* *niloticus*), Kobi fish and Tilapia (*Oreochromis niloticus*), dried small fish (*Rastrineobola argentea*), fish (*Clarias gariepinus*), fish powder (made from different fish species), smoked Barracuda fillet (*Sphyraena barracuda*), dried anchovies (*Stolephorus commersonnii*), Kapenta fish (*Limnothrissa miodon*), dried Catfish fillet (*Clarias gariepinus*).
	Prawns	Ground prawns (*Dendrobranch iata*), whole smoked prawns (*Penaeus vannamei*).
Spices (SPs)	Berbere ground pepper (*Capsicum*), fenugreek (*Trigonella foenum graecum*), turmeric (*Curcuma longa*), chilli powder mitmita (*Capsicum frutescens*), Banga spice (*Tetrapleura tetraptera*), Fasika spice (different spices mixture), African hot pepper powder (*Capsicum frutescens*), Cameroon pepper (*Piper guineense*), Suya kebab powder (blend of peanuts, chili, ginger, and other spices), ground fennel (*Foeniculum vulgare*), Najansa (*Ricinodendron*).	
Non-food items/additives (NFAs)	Kaun-Potash (group of potassium-bearing minerals), calabash—chalk, Calabar stone, or Nzu (aluminum Silicate Hydroxide), Chuna (calcium hydroxide).	

**Table 2:** Average concentrations and ranges (µg/g) of trace essential elements (TEEs) Cu, Fe, Mn, Se, Zn in dry weight of different foods: leaves (N = 22), tuber-crops (N = 6), Seed (N = 32), fruit (N = 20), legumes (N = 14), spices (N = 22), fish (N = 24), prawns (N = 4), gum Arabic (N = 2), Kaun-Potash-(N = 2), calabash chalk-Nzu (N = 2), and Chuna (N = 2).

No.	Subcategory (n)	Trace Essential Elements (TEEs)				
1	**Leaves (N =** **22)**	Cu	Fe	Mn	Se	Zn
	Mean (range)	7.9 (2.7–14.1)	298.8 (10.2–937.2)	94.4 (2.9–440.4)	0.2 (0.04–0.7)	19.4 (0.8–45)
2	**Tuber-crops (N = 6)**					
	Mean (range)	2.2 (0.5–4.4)	26.9 (14.2–49.2)	2.5 (1.2–4.2)	ND ^#^	4 (2.6–5.6)
3	**Seeds (N = 32)**					
	Mean (range)	4.7 (1.2–13.9)	31.7 (4.9–69.3)	11.3 (2.1–38.9)	0.3 (0.1–2.6)	11.6 (0.1–29.1)
4	**Fruits (N = 20)**					
	Mean (range)	7.6 (0.02–27.8)	54.1 (1.1–180)	10.5 (1.1–24.5)	1.3 (0.04–6.9)	10.01 (0.1–33.6)
5	**Legumes (N = 14)**					
	Mean (range)	6.5 (1.5–11.5)	53.1 (20.1–159.2)	17.2 (3–46.2)	0.1 (0.1–0.3)	7.4 (0.1–17.6)
6	**Spices (N = 22)**					
	Mean (range)	7.3 (1.2–14)	263 (5.7–892.7)	28.5 (2.1–73.5)	0.1 (0.04–0.3)	11.4 (0.1–40.4)
7	**Fish (N = 24)**					
	Mean (range)	9.4 (0.1–258.2)	85.8 (2.1–207.3)	26.1 (0.1–5.0)	1.3 (0.1–77.8)	29.5 (0.4–32.3)
8	**Prawns (N = 4)**					
	Mean (range)	37.6 (26.3–45.7)	129.5 (112.9–207.1)	5.7 (5.2–6.1)	1.8 (1.7–2.1)	24 (20.4–32.6)
9	**Gum (N = 2)**					
	Mean (range)	0.05 (0.04–0.05)	6.8 (0.1–13.4)	ND	0.01 (0.01–0.01)	0.06 (0.04–0.07)
10	**Kaun-Potash-(N = 2)**					
	Mean (range)	7.4 (6.8–7.9)	3227.6 (2922.8–3532.5)	83.8 (74.7–92.9)	0.4 (0.39–0.41)	6.7 (5.8–7.5)
11	**Calabash Chalk (Nzu) (N = 2)**					
	Mean (range)	12.2 (10.2–14.1)	555.6 (555.5–555.6)	12.4 (11.2–13.6)	0.9 (0.8–0.9)	9.5 (9.1–9.8)
12	**Chuna (N = 2)**					
	Mean (range)	2.6 (7.7–12)	150.8 (36.8–38.9)	46.1 (14–14.3)	0.5 (0.5–0.5)	2.5 (25.6–26)
**Mean concentration (µg/g) of the TEEs in the four food categories**						
**Food category**		Cu	Fe	Mn	Se	Zn
**1-Agricultural products**		4.8	78.6	22.7	0.3	8.7
**2-Spices**		7.3	263	28.5	0.1	11.4
**3-Fishery products**		23.5	107.7	15.9	1.6	26.8
**4-Non-food items/additives**		7.4	1311.3	47.4	0.6	6.2

^#^ ND = not detected.

**Table 3:** Calculated estimated dietary intake (EDI) mg/day for trace essential elements (TEEs).

Trace Essential Elements (TEEs), Estimated Dietary Intake (EDI) mg/Day						
No.	Subcategory	Cu	Fe	Mn	Se	Zn
1	**Leaves**	0.01	0.19	0.06	0.0001	0.01
2	**Tuber-crops**	0.08	1.02	0.09	ND	0.15
3	**Seeds**	0.69	4.66	1.66	0.04	1.70
4	**Fruits**	0.01	0.05	0.01	0.001	0.01
5	**Legumes**	0.17	1.42	0.46	0.003	0.20
6	**Gum Arabic (GA)**	0.002	0.204	ND	0.0003	0.002
7	**Spices**	0.01	0.33	0.04	0.0001	0.01
8	**Fish**	0.39	3.52	1.07	0.05	1.21
9	**Prawns**	1.54	5.31	0.23	0.07	0.98
10	**Kaun**	0.004	1.60	0.04	0.0002	0.00
11	**Calabash Chalk-Nzu**	0.20	11.10	0.20	0.02	0.20
12	**Chuna**	0.001	0.08	0.02	0.0003	0.001
**UL and RDI (mg/day)**		Cu	Fe	Mn	Se	Zn
**Upper intake limit (UL) mg/day ^a^**		10	45	11	0.4	40
**Recommended daily intake (RDI) mg/day ^a^**		0.9	8 & 18 *	1.8 & 2.3 *	0.06	8 & 11 *

^a^ [2]; * male and female.

**Table 4:** Percentages of recommended daily allowance (%RDA) for TEEs (Cu, Fe, Mn, Se, and Zn) in subcategories of foods and the mean of %RDA for the four food categories.

Trace Essential Element (%RDA)									
No.	Food Subcategory	Cu	Fe (Male)	Fe (Female)	Mn (Male)	Mn (Female)	Se	Zn (Male)	Zn (Female)
1	**Leaves**	0.6	2.4	1.1	2.7	3.4	<0.001	0.1	0.2
2	**Tuber-crops**	24.4	12.7	5.6	4.1	5.3	ND	1.4	1.9
3	**Seeds**	76.7	58.2	25.9	72.2	92.2	0.7	15.5	21.3
4	**Fruits**	0.8	0.6	0.3	0.4	0.6	<0.001	0.1	0.1
5	**Legumes**	19.4	17.8	7.9	20	25.6	<0.001	1.8	2.5
6	**Gum Arabic (GA)**	0.2	2.6	1.1	ND	ND	0.01	0.02	0.02
7	**Spices**	1	4.1	1.8	1.5	2	<0.001	0.1	0.2
8	**Fish**	42.8	44	19.5	46.5	59.5	0.9	11	15.1
9	**Prawns**	171.3	66.4	29.5	10.2	13	1.2	8.9	12.3
10	**Kaun**	0.4	20.2	9	1.8	2.3	0.003	0.03	0.04
11	**Calabash Chalk-Nzu**	27.1	138.9	61.7	10.8	13.8	0.3	1.7	2.4
12	**Chuna**	0.1	0.9	0.4	1	1.3	0.004	0.01	0.02
Average (%RDA) for TEEs									
	**Food** **Category**	**Cu**	**Fe (male)**	**Fe (female)**	**Mn (male)**	**Mn** **(female)**	**Se**	**Zn (male)**	**Zn (female)**
1	**Agricultural products**	20.3	15.7	7.0	16.6	21.2	0.1	3.1	4.3
2	**Spices**	1.0	4.1	1.8	1.5	2.0	0.0	0.1	0.2
3	**Fishery products**	107.1	55.2	24.5	28.4	36.3	1.1	10.0	13.7
4	**Non-food items/additives**	9.2	53.3	23.7	4.5	5.8	0.1	0.6	0.8

**Table 5:** Average concentrations and ranges (mg/g) of major essential elements (MEEs) Mg, K, Ca in dry weight of different foods: leaves (N = 22) tuber-crops (N = 6), seed (N = 32), fruits (N = 20), legumes (N = 14), spices (N = 22), fish (N = 24), prawns (N = 4), gum Arabic (N = 2), Kaun-Potash-(N = 2), calabash chalk-Nzu (N = 2), and Chuna (N = 2).

No.	Subcategory (n)	Major Essential Elements (MEEs)			
1	Leaves (N = 22)	Na	Mg	K	Ca
	Mean (mg/g)	2.3 (0.02–20.5)	2.9 (0.8–6.3)	17.2 (3.6–34.7)	12.4 (0.7–27.8)
2	Tuber-crops (N = 6)				
	Mean (mg/g)	0.3 (0.04–0.8)	0.3 (0.03–0.5)	4.6 (4.88.7)	0.4 (0.1–0.8)
3	Seeds (N = 30)				
	Mean (mg/g)	0.4 (0.01–6.4)	1.5 (0.2–4.0)	5.0 (0.9–14.7)	1.5 (0.01–8.8)
4	Fruits (N = 20)				
	Mean (mg/kg)	0.5 (0.01–2.8)	1.7 (0.4–4.8)	14.0 (0.5–20.7)	3.7 (0.2–11.7)
5	Legumes (N = 14)				
	Mean (mg/g)	0.04 (0.01–0.1)	1.4 (0.7–2.0)	8.4 (4.1–15.3)	0.7 (0.2–1.0)
6	Spices (N = 24)				
	Mean (mg/g)	11.2 (0.02–62.7)	1.9 (0.8–2.9)	14.6 (5.4–25.5)	3.3 (0.6–10.4)
7	Fish (N = 24)				
	Mean (mg/g)	14.6 (1.7–73.9)	1.5 (0.9–2.8)	10.4 (5.7–14.2)	15.3 (2.2–34.4)
8	Prawns (N = 4)				
	Mean (mg/g)	11.9 (9.6–13.8)	3.3 (3.1–3.3)	8.8 (7.6–9.8)	39.7 (35.1–45.5)
9	Gum Arabic (N = 2)				
	Mean (mg/g)	ND	2.0 (2.0–2.0)	5.9 (5.8–5.9)	6.2 (6.1–6.2)
10	Kaun-Potash-(N = 2)				
	Mean (mg/kg)	98.5 (85.8–111.3)	9.4 (8.3–10.6)	15.8 (15.1–16.5)	18.9 (17.0–20.7)
11	Calabash chalk-Nzu (N = 2)				
	Mean (mg/g)	0.1 (0.1–0.1)	2.0 (1.9–2.1)	8.9 (8.4–9.5)	0.2 (0.2–0.21)
12	Chuna (N = 2)				
	Mean (mg/g)	0.1 (0.1–0.1)	5.0 (5.0–5.0)	0.07 (0.05–0.09)	364.3 (360.7–368.0)
**Mean concentration (mg/g) of the MEEs in the four food categories**					
Food category		Na	Mg	K	Ca
1-Agricultural products		0.6	1.6	9.2	4.1
2-Spices		11.2	1.9	14.6	3.3
3-Fishery products		13.3	2.4	9.6	27.5
4-Non-food additives		32.9	5.5	8.3	127.8

**Table 6:** Calculated estimated dietary intake (EDI) mg/day for major essential elements (MEEs).

Major Essential Elements (MEEs), Estimated Dietary Intake (EDI) mg/Day					
No.	Subcategory	Na	Mg	K	Ca
**1**	** Leaves**	1.5	1.9	11.2	8.1
**2**	** Tuber-Plant**	11.3	11.8	175.3	13.6
**3**	** Seeds**	64.3	227.7	740.4	217.0
**4**	** Fruits**	0.5	1.6	13.3	3.5
**5**	** Legumes**	1.1	36.9	226.0	18.6
**6**	** Gum Arabic (GA)**	ND	61.2	176	185.3
**7**	** Spices**	13.9	2.3	18.1	4.0
**8**	** Fish**	599.6	63.3	425.3	628.5
**9**	** Prawns**	488.3	133.6	360.1	1627.1
**10**	** Kaun**	49.3	4.7	7.9	9.4
**11**	**Calabash Chalk-Nzu**	2.3	40.1	178.2	4.0
**12**	** Chuna**	0.1	2.5	0.04	182.2
**UL and RDI (mg/day)**		Na	Mg	K	Ca
**Upper intake limit (UL) mg/day ^a^**		2300	3500	-#	2500
**Recommended daily intake (RDI) mg/day ^a^**		1500	400 & 310 *	3400 & 2600 *	1000

^a^ [3]; available # No established UL; this is the highest adequate intake (AI) value. * male and female.

**Table 7:** Percentage of recommended daily allowance (%RDA) for MEEs (Na, Mg, K, and Ca) in subcategories of foods and mean of %RDA for the four food categories.

Major Essential Elements-(%RDA)							
No.	Subcategory	Na	Mg (Male)	Mg (Female)	K (Male)	K (Female)	Ca
**1**	** Leaves**	0.1	0.5	0.6	0.33	0.4	0.8
**2**	** Tuber-crops**	0.8	2.9	3.8	5.2	6.7	1.4
**3**	** Seeds**	4.3	56.9	73.4	21.8	28.5	21.7
**4**	** Fruits**	0.0	0.4	0.5	0.4	0.5	0.4
**5**	** Legume**	0.1	9.2	11.9	6.6	8.7	1.9
**6**	** Gum Arabic**	ND	15.3	19.7	5.2	6.8	18.5
**7**	** Spices**	0.9	0.6	0.7	0.5	0.7	0.4
**8**	** Fish**	40.0	15.8	20.4	12.5	16.4	62.9
**9**	** Prawns**	32.6	33.4	43.1	10.6	13.9	162.7
**10**	** Kaun**	3.3	1.2	1.5	0.2	0.3	0.9
**11**	** Calabash**	0.2	10.0	12.9	5.2	6.9	0.4
**12**	** Chuna**	0.0	0.6	0.8	0.0	0.0	18.2
**Average (%RDA) for MEEs**							
**Mean %RDA**		**Na**	**Mg (male)**	**Mg (female)**	**K (male)**	**K (female)**	**Ca**
**Agricultural products**		0.9	14.2	18.3	6.6	8.6	7.4
**Spices**		74.6	46.8	60.4	42.9	56.1	32.5
**Fishery products**		36.3	24.6	31.8	11.6	15.1	112.8
**Non-food additives**		1.1	3.9	5.1	1.8	2.4	6.5

## Data Availability

The original contribution presented in this study is included in the article. Further inquiries can be directed to the corresponding author.

## Abbreviations

The following abbreviations are used in this manuscript:

EDI	Estimated dietary intake
EEs	Essential elements
EI	Excess index
FPs	Fishery products
HI	Hazard index
HQ	Hazard quotient
MEES	Major essential elements
NFAs	Non-food items/additives
PBAPs	Plant-based agricultural products
RDA	Recommended daily allowance
SPs	Spices
TEEs	Trace essential elements
ULs	Upper intake limits
